# Isolation, Chemical Profile and Antimalarial Activities of Bioactive Compounds from *Rauvolfia caffra* Sond

**DOI:** 10.3390/molecules24010039

**Published:** 2018-12-21

**Authors:** Dorcas B. Tlhapi, Isaiah D. I. Ramaite, Teunis Van Ree, Chinedu P. Anokwuru, Taglialatela-Scafati Orazio, Heinrich C. Hoppe

**Affiliations:** 1 Department of Chemistry, University of Venda, Private Bag X5050, Thohoyandou 0950, South Africa; dorcastlhapi@gmail.com (D.B.T.); Teuns.VanRee@univen.ac.za (T.v.R.); anokwuruchi@gmail.com (C.P.A.); 2 Department of Pharmacy, University of Naples Federico II Via D. Montesano 49, 1-80131 Napoli, Italy; scatagli@unina.it; 3 Department of Biochemistry and Microbiology, Rhodes University, Grahamstown 6140, South Africa; H.Hoppe@ru.ac.za

**Keywords:** bioactive compounds, *Rauvolfia caffra* Sond, antiplasmodial activity

## Abstract

In this study, the chemical profile of a crude methanol extract of *Rauvolfia caffra* Sond was determined by ultra-performance liquid chromatography-mass spectrometry (UPLC-MS). Column chromatography and preparative thin layer chromatography were used to isolate three indole alkaloids (raucaffricine, *N*-methylsarpagine and spegatrine) and one triterpenoid (lupeol). The antiplasmodial activity was determined using the parasite lactate dehydrogenase (pLDH) assay. The UPLC-MS profile of the crude extract reveals that the major constituents of *R. caffra* are raucaffricine (*m*/*z* 513.2) and spegatrine (*m*/*z* 352.2). Fraction 3 displayed the highest antiplasmodial activity with an IC_50_ of 6.533 μg/mL. However, raucaffricine, isolated from the active fraction did not display any activity. The study identifies the major constituents of *R. caffra* and also demonstrates that the major constituents do not contribute to the antiplasmodial activity of *R. caffra*.

## 1. Introduction

In many countries worldwide medicinal plants remain the dominant form of medicine for the treatment and prevention of a wide range of diseases. Medicinal plants used as alternative drugs are indicative of the vital role that plants play in many developing countries, and are also sources of novel plant-derived constituents that could be leads for treatment of malaria and other diseases. Malaria is a major public health problem in the world, responsible for the death of millions, particularly in sub-Saharan Africa [1,2]. Today, the control of malaria has become gradually more complex due to the spread of drug-resistant parasites [3,4]. The WHO estimated that globally, about 80% of people with malaria live in rural areas [5]. In Africa, 22 million new cases of malaria are reported every year and about 8.6% deaths occur in the region due to malaria [2,6].

Treatment, management, and control of malaria are still a challenge to the medical community and safer, cheaper and more accessible alternatives are needed to efficiently limit the problem of this ailment [7]. Furthermore, the use of natural products for the treatment of malaria is becoming well-known due to the lesser side effects, accessibility and cost efficiency in comparison with conservative synthetic drugs. However, the number of plants with potential anti-malarial activities is limited and many of the purported anti-malarial properties have not been proven scientifically. Moreover, modes of action and active principles from most of these natural products are still unknown [8,9,10,11,12,13,14,15,16].

*Rauvolfia caffra* Sond is a plant species belonging to the family Apocynaceae [17]. *R. caffra* has been used widely for treatment of various conditions such as fever, swellings, abscesses, hepatitis, pneumonia, measles, skin lesions or itching rashes, cough, toothaches, sexually transmitted diseases, swollen legs, severe abdominal pains, abdominal disorders and wounds [18,19,20,21]. However, to the best of our knowledge, no in vivo or in vitro studies of potential applications of *R. caffra* in the treatment of these diseases have been reported. More generally, *Rauvolfia* species are commonly used in the treatment of malaria, diabetes, coughs, gastrointestinal disturbances, skin infections, hypertension, diarrhoea, dysentery, scabies, worm infections, parasitic and microbial infections. The ethnomedicinal uses make it one of the most important medicinal plants used in the suppression of skin diseases and opportunistic infections in HIV/AIDS patients in South Africa [22,23,24,25,26]. *R. caffra* Sond is rich in indole alkaloids, many of which have been isolated and identified [27,28]. The roots and stem bark extract possess high concentrations of reserpine, ajmaline and ajmalicine, which are known as antihypertensive and anti-inflammatory agents [29,30]. Furthermore, the extracts and derived alkaloids possess high antimicrobial activity due to the inhibition of certain redox pathways and other chemical processes in the bacterium cell [31]. These alkaloids also possess biological activities, such as antimalarial, antitumor and antidiabetic activity [29,30].

*R. caffra* also contains saponins, which have a wide range of biological actions, including antimicrobial, antiviral, antioxidant and cytotoxic properties [32]. *Rauvolfia caffra* has been reported to contain secondary metabolites that have medicinal and economic value. However, not much is known about the biological activities of the isolated compounds from *R. caffra*. Moreover, many of the reports on the biological activities have been limited to crude extracts. Against this backdrop, we decided to isolate compounds from the stem bark and evaluate their antimalarial activity. The UPLC-MS profile indicated that raucaffricine is the major compound, but it was not active despite good activity of the fraction from which it was isolated. This means that the major compound does not contribute to the antiplasmodial activity. Our results confirm the reported antiplasmodial activity of the crude methanol extracts from *Rauvolfia caffra* [29]. However, isolated compounds from this plant have not yet been studied for their antiplasmodial activity. Therefore, this study is the first survey to detect significant antiplasmodial activity of *R. caffra*.

## 2. Results and Discussion

### 2.1. Chemical Profile

The chemical profile (Figure S1) of the crude extract was obtained by ultra-performance liquid chromatography mass spectrometry (UPLC-MS). Two major peaks with molecular ions *m*/*z* 513.2 (5.21) and *m*/*z* 301.1 were observed in the crude extract. The peak with the molecular ion *m*/*z* 513.2 was identified as raucaffricine, whereas the molecular ion *m*/*z* 301.1 was not identified due to the fact that the compound was lost during column fractionation. The chromatogram illustrates that raucaffricine is a major constituent of *R. caffra* stem bark. The chemical profile of fraction F_3_ (Figure S2) revealed three major constituents with the molecular ions *m*/*z* 327.2, *m*/*z* 513.2 and *m*/*z* 349.2, where raucaffricine, with the molecular ion *m*/*z* 513.2 (5.18) was found to be the major constituent of Fraction F_3_. The compounds with molecular ions *m*/*z* 327.2 and *m*/*z* 349.2 could not be isolated from Fraction F_3_. Furthermore, the chemical profile of Fraction F_5_ (Figure S3) revealed two major constituents, with the molecular ions *m*/*z* 325.2 and *m*/*z* 335.1, respectively, where the molecular ion *m*/*z* 325.2 was identified as that of spegatrine, whereas the compound with molecular ion *m*/*z* 335.1 was not isolated. Spegatrine with molecular ion *m*/*z* 325.2 was identified as a major constituent of fraction F_5_ but not a major constituent of the crude extract.

### 2.2. Isolation and Purification of Compounds

**Figure 1:**
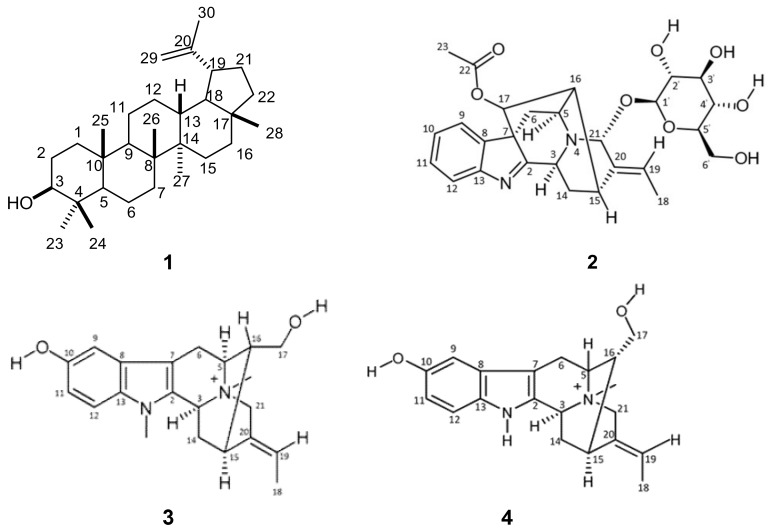
Isolated triterpenoid **1** and indole alkaloids **2**–**4**.

The crude methanol extract was subjected to column chromatography using silica gel to obtain five fractions (F_1–5_). Lupeol (**1**, Figure 1) was isolated as a white powder from a polar fraction (F_1_) by repeated silica gel and reversed phase chromatography. A combination of nuclear magnetic resonance (1D- and 2D-NMR), infrared spectroscopy (IR) and high resolution mass spectrometric (HRMS) analysis was used for the structural elucidation of isolated compounds. Lupeol has the potential to act as an anti-inflammatory, anti-arthritic, anti-microbial, anti-poliferative and anti-protozoal [33]. Raucaffricine (**2**) was isolated as a brown amorphous solid from Fraction F_3_ (0.5 g) after purification by semi-preparative HPLC. Fraction F_4_ was subjected to preparative TLC (reversed phase) to yield *N*-methylsarpagine (**3**), a brown amorphous solid, and Fraction F_5_ was purified using semi-preparative HPLC to yield spegatrine (**4**), a brown amorphous solid. According to a literature survey, the biological activities of raucaffricine, *N*-methylsarpagine and spegatrine have never been evaluated. Therefore, that makes our study the first to test their biological activities.

The IR spectrum of lupeol (**1**) had a band corresponding to hydroxyl (3000 cm^−1^, O-H), and ^13^C-NMR results revealed the presence of 30 carbon signals. The ^1^H-NMR spectrum revealed the presence of six tertiary methyl protons at δ_H_ 0.77 ppm (3H, s), δ_H_ 0.86 ppm (3H, s), δ_H_ 0.97 ppm (3H, s), δ_H_ 1.0 ppm (3H, s), δ_H_ 1.04 ppm (3H, s) and δ_H_ 1.70 ppm (3H, s), corresponding to protons at positions 24, 25, 27, 23, 26 and 30, respectively. A multiplet of one proton at δ_H_ 2.38 ppm (1H, m) assigned to 19-H is characteristic of lupeol. The 3-H proton displayed a multiplet at δ_H_ 3.18 ppm (1H, m) while a pair of broad singlets at δ_H_ 4.7 ppm (1H, s) and δ_H_ 4.6 ppm (1H, s) was indicative of olefinic protons at (29a-H and 29b-H). These assignments are in good agreement with the structure of lupeol (**1**).

Raucaffricine (**2**) exhibited a [M]^+^ ion peak at *m*/*z* 513.2237 which matched the molecular formula C_27_H_32_N_2_O_8_. The IR showed characteristic absorption frequencies at 3312.90, 2942.44 and 2831.65 cm^−1^ typical of the O-H, C-H and C-H bond vibrations, respectively. This was supported by the appearance of 27 carbon signals in its ^13^C-NMR spectrum, including six quaternary carbons, fifteen methines, four methylenes and two methyl groups at δ 21.32 (Q, C-23) and 13.28 (Q, C-18), respectively. The ^1^H-NMR spectrum showed an *ortho*-disubstituted aromatic ring with δ_H_ 7.57 to 7.2 ppm, which included two sets of doublets at δ_H_ 7.55 and 7.49 ppm corresponding to protons at positions 9 and 12, a doublet of doublets at δ_H_ 7.39 ppm corresponding to a proton at position 10 and another doublet of doublets at δ_H_ 7.23 ppm corresponding to a proton at position 11. The spectrum also showed the existence of an ethylidene group with a doublet at δ_H_ 1.67 ppm assigned to position 18 and a quartet at δ_H_ 5.65 ppm assigned to a proton at position 19. The ^1^H-NMR also revealed several other important features, including one methyl signal at δ_H_ 2.16 (s) corresponding to OCOCH_3_ and three methylene signals at δ_H_ 4.42 ppm, δ_H_ 1.87 and δ_H_ 1.77 ppm, and δ_H_ 2.68 and δ_H_ 1.45 ppm, corresponding to 6′-H, 14-H (α and β), and 6-H (β and α), respectively. Moreover, a glucopyranosyl moiety was indicated by the signal of the anomeric proton at δ_H_ 5.03 (1H, d, *J* = 4.4 Hz, 1′-H) and in agreement with carbon signals at δ_C_ 99.32 (CH, C-1′), 77.48 (CH, C-3′, C-5**^′^**), 74.26 (CH, C-2′), 70.58 (CH, C-4′) and 61.56 (CH_2_, C-6′). The complete assignment of the ^1^H-NMR and ^13^C-NMR chemical shifts was done using 2D-NMR (COSY, HSQC and HMBC) spectroscopic techniques. These results enabled us to confirm the ^1^H-NMR and ^13^C-NMR chemical attributions of raucaffricine as previously reported [34].

The ^13^C-NMR of *N*-methylsarpagine (**3**) showed 21 non-equivalent carbon signals. The ^1^H-NMR showed the presence of signals of three aromatic protons which included a doublet at δ_H_ 7.11 ppm (1H, d, *J* = 8.6 Hz), corresponding to a proton at position 12, another doublet at δ_H_ 6.77 ppm (1H, d, *J* = 2.0 Hz) corresponding to a proton at position 9 and a doublet of doublets at δ_H_ 6.68 ppm (1H, dd, *J* = 22. Hz and *J* = 8.9 Hz) corresponding to a proton at position 11. The ^1^H-NMR also showed the existence of an ethylidene group with a doublet at δ_H_ 1.63 ppm assigned to position 18 and a quartet at δ_H_ 5.59 ppm assigned to a proton at position 19. The ^1^H-NMR also displayed an exchangeable hydroxyl proton at δ_H_ 1.82 ppm, attached to C-10 (δ_C_ 150.91) and two methyl groups corresponding to N_1_-Me at δ_H_ 3.28 ppm and N^+^_4_-Me at δ_H_ 3.0 ppm. Total assignment was done by a close examination of the 1D-NMR (^1^H-NMR and ^13^C-NMR), 2D-NMR (COSY, HSQC and HMBC), UPLC-MS and literature data.

Spegatrine (**4**) revealed a [M + H]^+^ ion peak at *m*/*z* 325.1912 in its HRMS spectrum, suggesting the molecular formula C_20_H_25_N_2_O_2_^+^. The ^13^C-NMR displayed 20 non-equivalent carbon signals, which was consistent with the HRMS result. The ^1^H-NMR showed an active hydroxyl proton at δ 1.97 ppm, attached to C-10 (δ_C_ 150.93). The ^1^H-NMR was further characterized by a doublet at δ_H_ 7.24 ppm (1H, d, *J* = 8.8 Hz), corresponding to a proton at position 12, another doublet at δ_H_ 6.90 ppm (1H, d, *J* = 2.0 Hz) corresponding to a proton at position 9 and a doublet of doublets at δ_H_ 6.78 ppm (2H, dd, *J* = 2 Hz and *J* = 2.4 Hz) corresponding to a proton at position 11. Furthermore, the ^1^H-NMR showed an ethylidene group at δ_H_ 1.74 ppm (3H, d, *J* = 6.8 Hz) associated with position 18 and a quartet at δ_H_ 5.68 ppm (1H, q, *J* = 6.8 Hz) assigned to a proton at position 19. A N^+^-Me group at δ_H_ 3.12 ppm (s) which is a strong electron withdrawing group let the 21-H protons appear at δ_H_ 4.45 ppm (1H, d AB, *J* = 15.6 Hz) and at δ_H_ 4.23 ppm (1H, d AB, *J* = 15.6 Hz), respectively. Another signal shifted downfield was found at δ_H_ 3.58 ppm (1H, d, *J* = 7.6 Hz) corresponding to two protons at C-17 (δ_C_ 62.40). This condition may be due to the presence of a hydroxyl group attached to C-17 (δ_C_ 62.40). Total assignment was done by a close examination of the 1D-NMR (^1^H-NMR and ^13^C-NMR), 2D-NMR (COSY, HSQC and HMBC), UPLC-MS and literature data.

### 2.3. Antiplasmodial Activity

**Figure 2:**
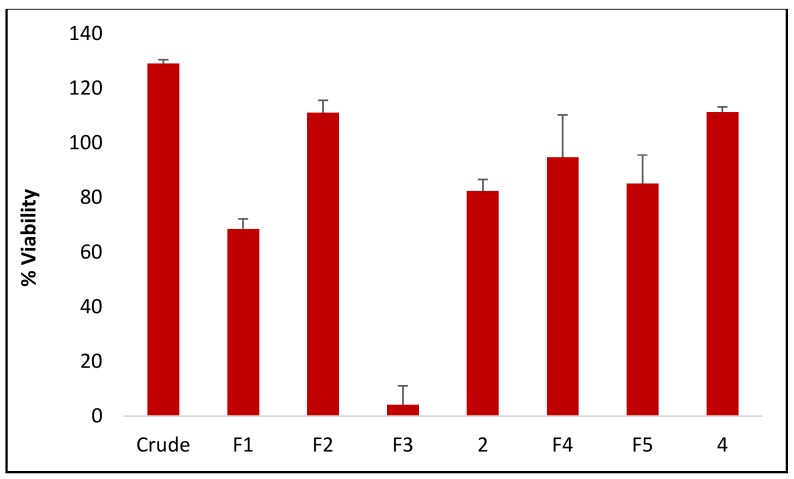
Antimalarial activity of crude, fractions, raucaffricine (**2**) and spegatrine (**4**) expressed as % parasite viability ± SD.

The highest antiplasmodial activity was found in fraction F_3_ with a viability of 4.149 ± 6.979% (Figure 2) and IC_50_ value of 6.533 μg/mL. The reference drug chloroquine showed an IC_50_ value of 0.032 µg/mL, and was higher in activity than F_3_. The major compound raucaffricine, which was isolated from F_3_ was not active. This result shows that the major constituent does not contribute to the antiplasmodial activity of *R. caffra*. Spegatrine also did not display any activity.

**Figure 3:**
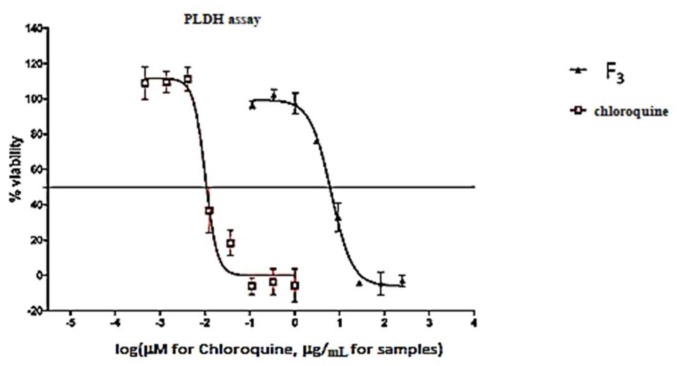
Dose-response curve for antimalarial assay.

As shown in Figure 3, Fraction F_3_ at a concentration 250 μg/mL decreased the viability of *Plasmodium falciparum* (4.149 ± 6.979%) with an IC_50_ value of 6.533 μg/mL, whereas the reference drug chloroquine showed an IC_50_ value of 0.032 µg/mL.

## 3. Materials and Methods

### 3.1. General Experimental Procedure

All chemicals used were analytical grade purchased from Sigma-Aldrich (Darmstadt, Germany). Silica gel (0.063–0.2 mm) or Sephadex LH20 was used as stationary phases and solvent mixtures described below were used as mobile phase in the chromatographic separations. Thin layer chromatography plates packed with silica gel (normal or reversed phase), were used to locate major components of the fractions.

#### 3.1.1. High-Resolution Mass Spectrometry

A Waters Synapt G2 Quadrupole time-of-flight (QTOF) mass spectrometer (MS) connected to a Waters Acquity ultra-performance liquid chromatograph (UPLC) (Waters, Milford, MA, USA) was used for direct injection high resolution mass spectrometric analysis. 1 μL of sample was injected into a stream of 60% acetonitrile/40% 0.1% formic acid in water. This conveyed the sample directly to the QTOF mass spectrometer where data was acquired using both positive and negative electrospray ionisation. The following MS settings were used: cone voltage of 15 V, desolvation temperature of 275 °C, desolvation gas at 650 L/h, and the rest of the MS settings optimized for best resolution and sensitivity.

#### 3.1.2. Infrared Spectroscopy

ATR Infrared (IR) spectra were recorded on an Alpha FTIR spectrometer (Bruker, Fällanden, Switzerland).

#### 3.1.3. NMR Spectroscopy

^1^H- and ^13^C-nuclear magnetic resonance (NMR) spectra were recorded at 400 MHz and 100 MHz, respectively, with an Avance 400 spectrometer (Bruker) using residual undeuterated solvent as the internal standard.

#### 3.1.4. Liquid Chromatography-Mass Spectrometry (LCMS) Analysis

The instrumentation used for ultra-performance liquid chromatography MS (UPLC-MS) was identical to that described above, except for the inclusion of a Waters BEH C_18_, 2.1 × 100 mm, 1.7 μm column into the flow stream. This facilitated separation of compounds prior to data acquisition using a photodiode array detector (PDA), followed by the QTOF MS where data was acquired by scanning from *m*/*z* 150 to 1500 *m*/*z* in resolution mode as well as in MSE mode. In MSE mode two channels of MS data were acquired, one at a low collision energy (4 V) and the second using a collision energy ramp (40−100 V) to obtain fragmentation data as well. Leucine enkephalin was used as lock mass (reference mass) for accurate mass determination and the instrument was calibrated with sodium formate. An injection volume of 3 μL was used and the mobile phase consisted of 0.1% formic acid (solvent A) and acetonitrile containing 0.1% formic acid as solvent B.

### 3.2. Plant Collection and Preparation

The stem bark of *Rauvolfia caffra* Sond was collected at the University of Venda Campus (Thohoyandou, Limpopo, South Africa) in January 2016. Botanical identification was provided by Prof. Tshisikhawe, a botanist in the Department of Botany at the University of Venda, and a voucher specimen (BD 001) was deposited. The plant samples were air-dried for two months and the dry samples were ground to fine powder using an industrial blender (mill).

### 3.3. Extraction of Plant Materials

About 1.7 kg ground stems of *R. caffra* were soaked with 2 L methanol for 48 h at room temperature. The methanol extract was filtered, and then concentrated using a rotary evaporator to obtain 49.2 g of dried extracts. The crude methanol extract (49.2 g) was subjected to column chromatography over silica gel.

The extract was eluted initially with hexane and the polarity was gradually increased with ethyl acetate and finally methanol, yielding 17 fractions. Fractions with similar TLC profile were combined and concentrated to dryness on a rotary evaporator giving a total of 8 fractions coded as F_A_–F_H_. F_A_ was obtained with hexane/ethyl acetate (70:30); F_B_, F_C_ were obtained with hexane/ethyl acetate (30:70); F_D_ was obtained with ethyl acetate (100%); F_E_, F_F_ were obtained with ethyl acetate/methanol (70:30); and F_G_, F_H_ were obtained with ethyl acetate/methanol (30:70). The collected fractions were monitored on TLC plates.

### 3.4. Isolation and Purification of Compounds

Fractions F_B_, F_E_, F_F_ and F_G_ were further fractionated using column chromatography since they contained a large amount of material compared to fractions F_A_, F_C_, F_D_ and F_H_.

Fraction F_B_ (1.42 g) was subjected to Sephadex LH-20 column chromatography; the column was eluted with CH_2_Cl_2_/MeOH (50:50) followed by an increasing gradient of CH_2_Cl_2_/MeOH (up to 10:90) to obtain F_1_ (1 g). Fraction F_E_ (4 g) was subjected to silica gel column chromatography; the column was eluted using CH_2_Cl_2_/MeOH (50:50) followed by an increasing gradient of CH_2_Cl_2_/MeOH (up to 10:90) to obtain 2 subfractions, F_2_ (1.51 g) and F_3_ (1.55 g). Fraction F_F_ (4 g) was also subjected to silica gel column chromatography and the column was eluted using *n*-C_6_H_12_/EtOAc (50:50) followed by an increasing gradient of *n*-C_6_H_12_/EtOAc (up to 30:70) to obtain 1 subfraction, F_4_ (3 g). Fraction F_G_ (5.6 g) was subjected to Sephadex LH-20 column chromatography; the column was eluted using CH_2_Cl_2_/MeOH (50:50) followed by an increasing gradient of CH_2_Cl_2_/MeOH (up to 10:90) to obtain F_5_ (5 g). The collected fractions were monitored on TLC plates. The thin layer chromatograms were developed in a solvent system of ethyl acetate/methanol/water (EMW 81:11:8). A natural product staining solution (1 g methanolic diphenylboric acid, 100 mL methanol, 5 mL PEG 400 and 95 mL ethanol) was used to visualize compounds on a TLC plate.

F_1_ (0.5 g) was subjected to preparative TLC (normal phase) to obtain compound **1** (0.230 g). Fraction F_2_ (0.5 g) was also subjected to preparative TLC (reversed phase) to obtain fraction F_2a_ (0.2102 g). Fraction F_3_ (0.5 g) was further purified using semi-preparative HPLC to yield compound **2** (0.019 g). Fraction F_4_ (0.5 g) was also subjected to preparative TLC (reversed phase) to obtain compound **3** (0.1238 g). F_5_ (1 g) was further purified using semi-preparative HPLC to obtain compound **4** (0.086 g).

*Lupeol* (**1**): IR: 3000 cm^−1^ (O-H). ^1^H-NMR (400 MHz, CD_3_OD): δ_H_ 4.7 (1H, s, 29a-H), 4.6 (1H, s, 29b-H), 3.18 (1H, m, 3-H), 2.38 (1H, m, 19-H), 1.91 (1H, m, 21-H), 1.70 (3H, s, 30-H), 1.60 (1H, m, 2-H), 1.39 (1H, m, 18-H), 1.30 (1H, m, 9-H), 1.04 (3H, s, 26-H), 1.0 (3H, s, 23-H), 0.97 (3H, s, 27-H), 0.86 (3H, s, 25-H), 0.77 (3H, s, 24-H) ppm. ^13^C-NMR (CD_3_OD): δ_C_ 150.95 (C-20), 108.54 (C-29), 78.27 (C-3), 55.48 (C-5), 50.64 (C-9), 49.16 (C-18), 42.17 (C-14), 40.53 (C-8), 38.67 (C-4), 38.54 (C-1), 38.17 (C-13), 37.00 (C-10), 36.91 (C-16), 34.21 (C-7), 30.43 (C-21), 29.52 (C-23), 29.27 (C-2), 27.19 (C-15), 25.54 (C-12), 22.79 (C-11), 20.70 (C-30), 18.17 (C-6), 18.03 (C-28), 15.32 (C-26), 14.69 (C-24), 13.67 (C-27) ppm.

*Raucaffricine* (**2**): IR: ν_O-H_ at 3421.5 cm^−1^ (O-H) and ν_C=O_ at 1662.0 cm^−1^ (C=O). ^1^H-NMR (400 MHz, DMSO-*d*_6_): δ_H_ 7.55 (1H, d, *J* = 7.6 Hz, 9-H), 7.49 (1H, d, *J* = 7.2 Hz, 12-H), 7.39 (1H, dd, *J* = 7.6 Hz and *J* = 7.6 Hz, 10-H), 7.23 (1H, dd, *J* = 7.2 Hz and *J* = 7.6 Hz, 11-H), 5.65 (1H, q, *J* = 6.8 Hz, 19-H), 5.17 (1H, s, 21-H), 5.03 (1H, d, *J* = 4.4 Hz, 1′-H), 4.57 (1H, s, 17-H), 4.42 (2H, dd, *J* = 7.6 Hz and *J* = 8.8 Hz, 6′-H), 3.9–3.6 (1H, m, 5′-H), 3.18 (1H, dd, *J* = 5.6 Hz and *J* = 6 Hz, 5-H), 3.08 (1H, m, 15-H), 2.68 (1H, dd, *J* = 4.8 Hz and 4.4 Hz, 6-H_β_), 2.34 (1H, dd, *J* = 6 and *J* = 6.4 Hz, 16-H), 2.16 (3H, s, OCOCH_3_), 1.87 (1H, dd, *J* = 4 Hz and *J* = 12 Hz, 14-H_α_), 1.77 (1H, dd, *J* = 4.8 Hz and *J* = 4.8 Hz, 14-H_β_), 1.67 (3H, d, *J* = 11.6 Hz, 18-CH_3_), 1.45 (1H, d, *J* = 6.8 Hz, 6-H_α_) ppm. ^13^C-NMR (DMSO-*d*_6_): δ_C_ 184.42 (C-2), 170.13 (C-22), 156.86 (C-13), 137.94 (C-20), 137.09 (C-8), 127.06 (C-11), 125.81 (C-10), 124.29 (C-9), 122.76 (C-19), 120.85 (C-12), 99.32 (C-1′), 88.22 (C-21), 77.48 (C-3′,5′), 77.21 (C-17), 74.26 (C-2′), 70.58 (C-4′), 65.19 (C-7), 61.56 (C-6′), 55.24 (C-5), 50.45 (C-3), 48.49 (C-16), 37.32 (C-6), 27.47 (C-15), 24.67 (C-14), 21.32 (C-23), 13.28 (C-18) ppm. HRMS [M]^+^: *m*/*z* 513.2241; calcd. for C_27_H_32_N_2_O_8_: 513.2237.

*N-Methylsarpagine* (**3**): IR: 3312.90 (O-H), 2942.44 (C-H) and 2831.65 cm^−1^ (C-H). ^1^H-NMR (400 MHz, CD_3_OD): δ_H_ 7.11 (1H, d, *J* = 8.6 Hz, 12-H), 6.77 (1H, d, *J* = 2.0 Hz, 9-H), 6.68 (1H, dd, *J* = 8.9, 2.2 Hz, 11-H), 5.59 (1H, q, *J* = 6.8 Hz, 19-H), 4.41 (1H, d AB, *J* = 15.6 Hz, 21-H_β_), 4.16 (1H, d AB, *J* = 15.6 Hz, 21-H_α_), 3.48 (2H, d, *J* = 7.2 Hz, 17-H), 3.28 (3H, s, N-CH_3_), 3.15 (1H, dd, *J* = 12.4, 4.8 Hz, 6-H_β_), 3.0 (3H, s, N^+^-CH_3_), 2.95 (1H, dd, *J* = 2.0, 10.4 Hz, 15-H), 2.9 (1H, d, *J* = 15.2 Hz, 6-H_α_), 2.4 (1H, dd, *J* = 11.6, 10.8 Hz, 14-Hα), 2.12–2.0 (2H, m, 16-H + 14-H_β_), 1.82 (s, OH), 1.63 (3H, d, *J* = 6.7 Hz, 18-H) ppm. ^13^C-NMR (CD_3_OD): δ_C_ 150.91 (C-10), 132.10 (C-20), 131.68 (C-13), 127.67 (C-8), 126.83 (C-2), 120.72 (C-19), 112.34 (C-12), 111.75 (C-11), 102.14 (C-9), 99.77 (C-7), 65.45 (C-5), 64.39 (C-17), 62.40 (C-21), 61.07 (C-3), 43.63 (C-16), 32.01 (C-14), 26.01 (C-15), 23.87 (C-6), 11.57 (C-18) ppm.

*Spegatrine* (**4**): IR: 3352.1 cm^−1^ (O-H) and 1638.8 cm^−1^ (N-H). ^1^H-NMR (400 MHz, CD_3_OD): δ_H_ 7.24 (1H, d, *J* = 8.8 Hz, 12-H), 6.90 (1H, d, *J* = 2.0 Hz, 9-H), 6.78 (1H, dd, *J* = 2.2, 8.8 Hz, 11-H), 5.68 (1H, q, *J* = 6.8 Hz, 19-H), 4.45 (1H, d AB, *J* = 15.6 Hz, 21-H_α_), 4.23 (1H, d AB, *J* = 15.6 Hz, 21-H_β_), 3.58 (2H, d, *J* = 7.6 Hz, 17-H), 3.27 (1H, dd, *J* = 12.4, 4.8 Hz, 6-H_β_), 3.15 (3H, s, N^+^-CH_3_), 3.12 (1H, dd, *J* = 2.0, 10.4 Hz, 15-H), 3.04 (1H, d, *J* = 17.2 Hz, 6-H_α_), 2.54 (1H, dd, *J* = 11.6, 10.8 Hz, 14-Hα), 2.23–2.13 (2H, m, 16-H + 14-H_β_), 1.97 (s, OH), 1.74 (3H, d, *J* = 6.8 Hz, 18-H) ppm. ^13^C-NMR (CD_3_OD): δ_C_ 150.93 (C-10), 132.10 (C-20), 131.66 (C-13), 127.68 (C-8), 126.83 (C-2), 120.71 (C-19), 112.35 (C-12), 111.73 (C-11), 102.13 (C-7), 99.77 (C-9), 65.46 (C-5), 64.38 (C-21), 62.40 (C-17), 61.08 (C-3), 46.67 (N^+^- CH_3_), 43.62 (C-16), 32.00 (C-14), 26.01 (C-15), 23.87 (C-6), 11.56 (C-18) ppm. HRMS [M]^+^: *m*/*z* 325.1912; calcd. for C_20_H_25_N_2_O_2_^+^: 325.1911.

### 3.5. Antiplasmodial Activity

Malaria parasites (*Plasmodium falciparum* chloroquine-sensitive strain 3D7, obtained from the Malaria Research and Reference Reagent Resource Center, Manassas, VA, USA) were maintained in RPMI 1640 medium containing 2 mM l-glutamine and 25 mM Hepes (Lonza Bioscience, Allendale, NJ, USA). The medium was further supplemented with 5% Albumax II, 20 mM glucose, 0.65 mM hypoxanthine, 60 µg/mL gentamycin and 2–4% hematocrit human red blood cells. The parasites were cultured at 37 °C under an atmosphere of 5% CO_2_, 5% O_2_ and 90% N_2_ in sealed T75 culture flasks. For screening compounds against malaria parasites, 20 μM for pure compounds (**2** and **4**) or 25 μg/mL of the crude extract and fractions F_1_, F_2_, F_3_, F_4_ and F_5_ were added to parasite cultures (2% parasitaemia, 1% hematocrit) in 96-well plates and incubated for 48 h at 37 °C in a sealed chamber suffused with the above gas mixture. After 48 h the plates were removed from the incubator and parasite levels in the individual wells determined using the parasite lactate dehydrogenase (pLDH) assay [35]. 20 µL culture was removed from each well and mixed with 100 µL Malstat solution (55 mM Tris, 0.22 M L-lactic acid, 0.17 mM acetylpyridine adenine dinucleotide [APAD], 0.2% [*v*/*v*] Triton X-100, pH 9.0) and 25 µL NBT/PES solution (1.96 mM nitrotetrazolium blue chloride, 0.24 mM phenazine ethosulphate) in a fresh 96-well plate. After incubation for 30 min, colour development was measured as absorbance at 620 nm (Abs_620_) in a Spectramax M3 plate reader (Molecular Dynamics Inc., Chatsworth, CA, USA). After subtracting background readings obtained from control wells (uninfected red blood cells), Abs_620_ values were converted to % parasite viability relative to untreated control cultures. For dose-response analysis, the above procedure was repeated by incubating parasite cultures in 96-well plates with 3-fold serial dilutions of the crude extract, fractions (F_1_, F_2_, F_3_, F_4_ and F_5_) and compounds **2** and **4**. Log (compound concentration) was plotted against % parasite viability and IC_50_ values calculated by non-linear regression using GraphPad Prism (GraphPad Software, San Diego, CA, USA). Chloroquine (an anti-malarial drug) was used as a positive control drug standard for antimalarial activity.

## 4. Conclusions

Four compounds were isolated from the stem bark methanol extract of *R. caffra.* Raucaffricine, a rare glycoalkaloid of the monoterpenoid indole class and spegatrine, an indole alkaloid isolated for the first time from *Rauvolfia verticillata*. This is the first report of the isolation of spegatrine from *R. caffra*. Lupeol, a pentacylic triterpenoid was previously isolated from various medicinal plants while *N*-methylsarpagine, an indole alkaloid, was first isolated from *Rauvolfia vomitoria*. This is the first report of the isolation of lupeol and *N*-methylsarpagine from the genus *Rauvolfia* and *R. caffra* species. The UPLC-MS chromatogram indicated that raucaffricine is a major alkaloid from *R. caffra* but did not contribute to the antiplasmodial activity of the fraction from which it was isolated.

## Supplementary Materials

The supplementary materials are available online.
