# Supplementary material for: Isolation, Chemical Profile and Antimalarial Activities of Bioactive Compounds from Rauvolfia caffra Sond
Source: Molecules. 2018 Dec 21;24(1):39. doi: 10.3390/molecules24010039 (PMC6337319; doi:10.3390/molecules24010039)
Supplement: Supplementary file 1 [file molecules-24-00039-s001.pdf]

Supplementary information

Isolation, chemical profile and antimalarial activities of bioactive compounds from *Rauvolfia caffra* Sond

Dorcas B Tlhapi [1], Isaiah D.I Ramaite [1,*], Teunis van Ree [1], Chinedu P Anokwuru [1], Taglialatela-Scafati Orazio [2] and Heinrich C Hoppe [3]

[1.] Department of Chemistry, University of Venda, Thohoyandou, Private Bag X5050, 0950, South Africa; dorcastlhapi@gmail.com; Isaiah.Ramaite@univen.ac.za; Teuns.VanRee@univen.ac.za, , anokwuruchi@gmail.com

[2.] Department of Pharmacy, University of Naples Federico II Via D. Montesano 49, 1-80131, Napoli (Italy); scatagli@unina.it

[3.] Department of Biochemistry and Microbiology, Rhodes University, Grahamstown, 6140 South Africa; H.Hoppe@ru.ac.za

* Correspondence: Isaiah.Ramaite@univen.ac.za; Tel.: +27(0)15-962-8262

Academic Editors:

Received: date; Accepted: date; Published: date

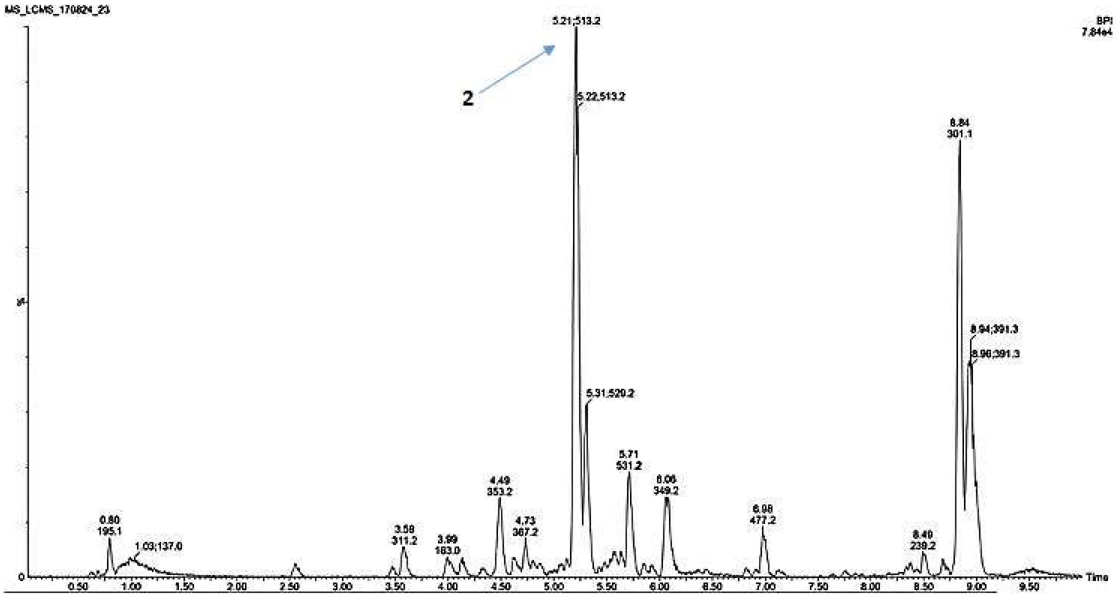

Figure S1. UPLC-MS of the crude extract.

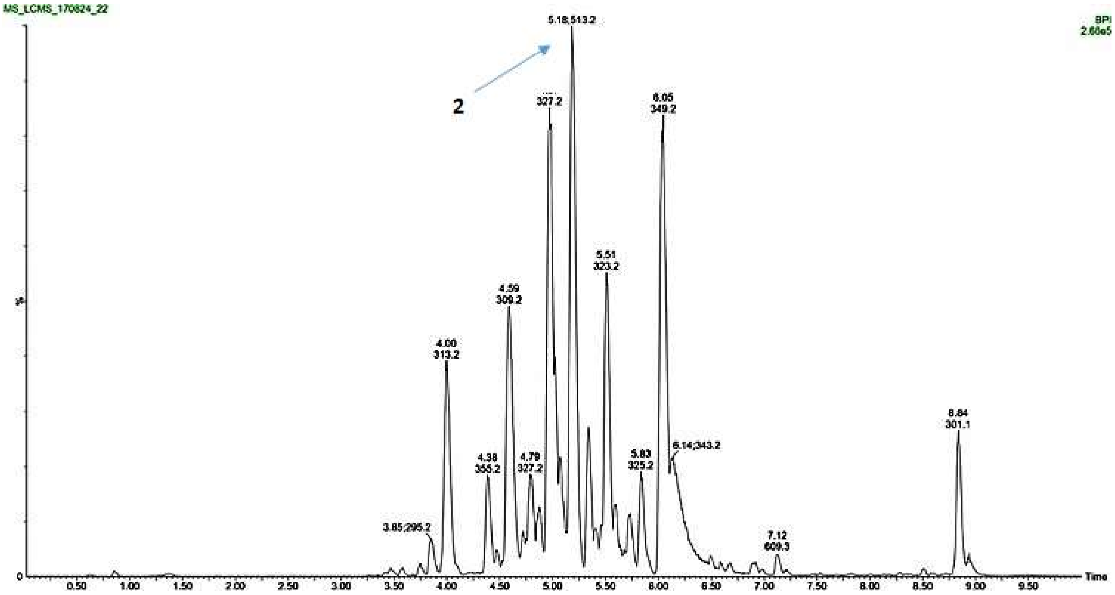

Figure S2. UPLC-MS of Fraction F3.

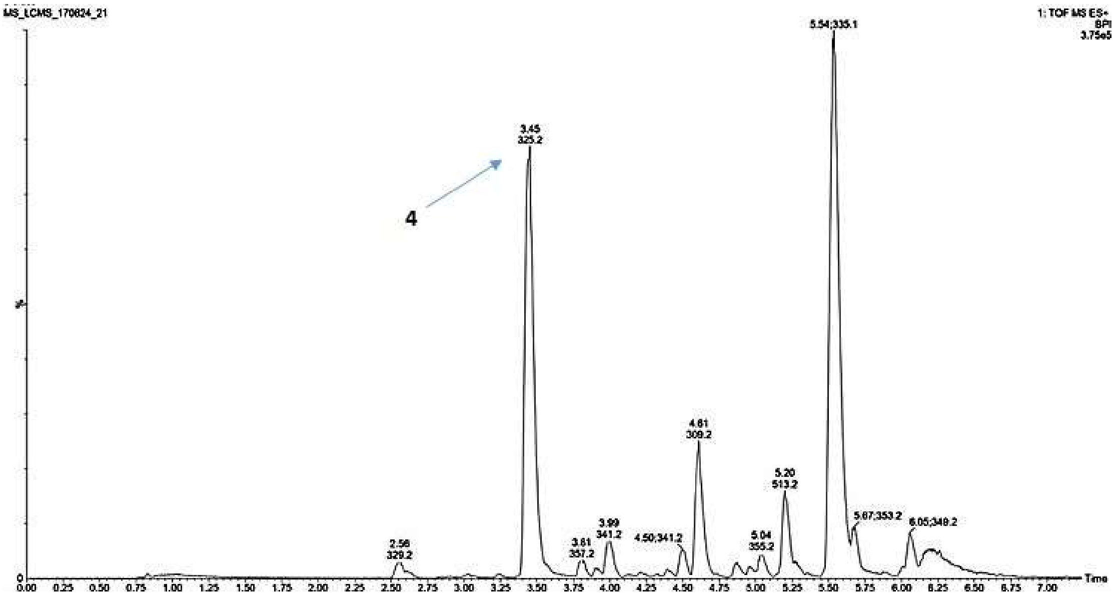

Figure S3. UPLC-MS of Fraction F5.

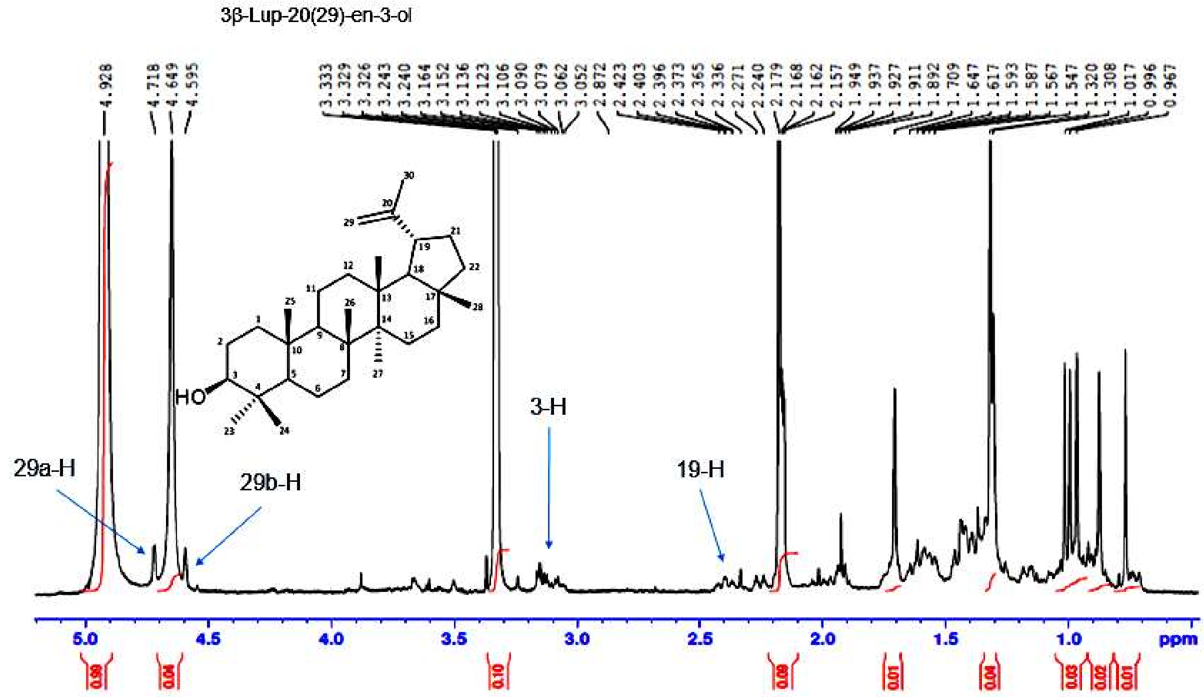

Figure S4. Expanded ^{1}H-NMR spectrum of lupeol (**1**).

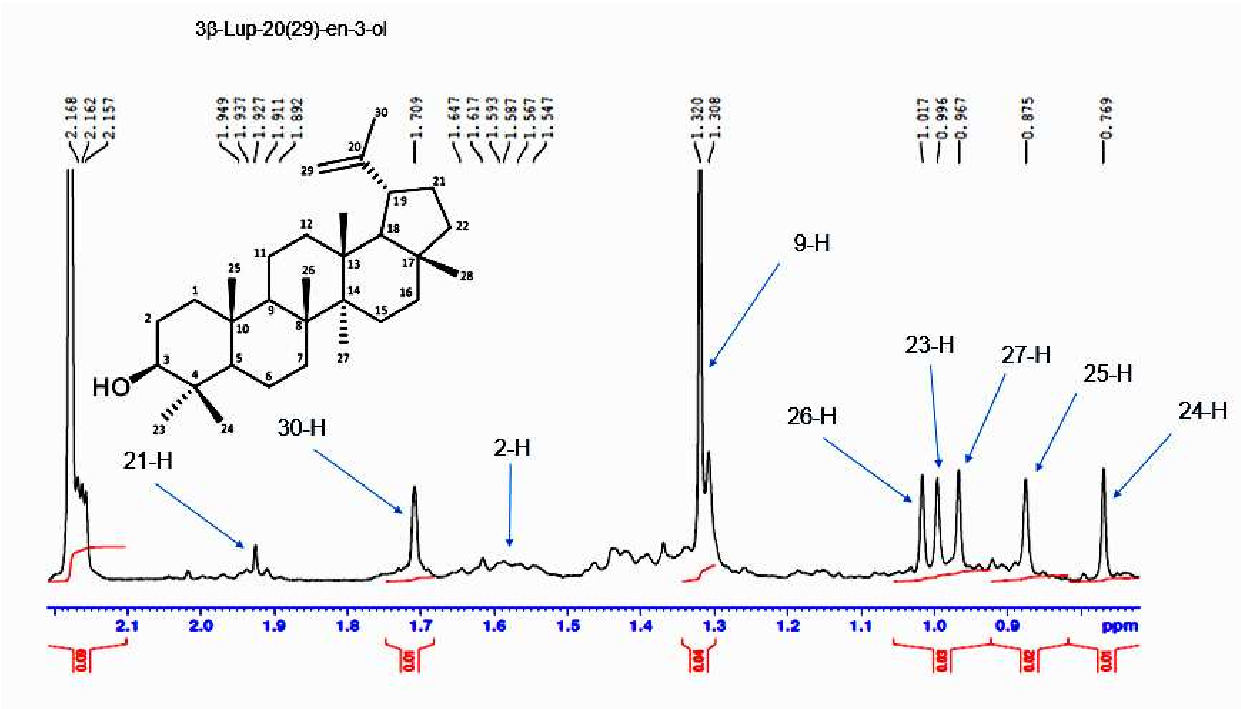

Figure S5. Expanded ^{1}H-NMR spectrum of lupeol (**1**).

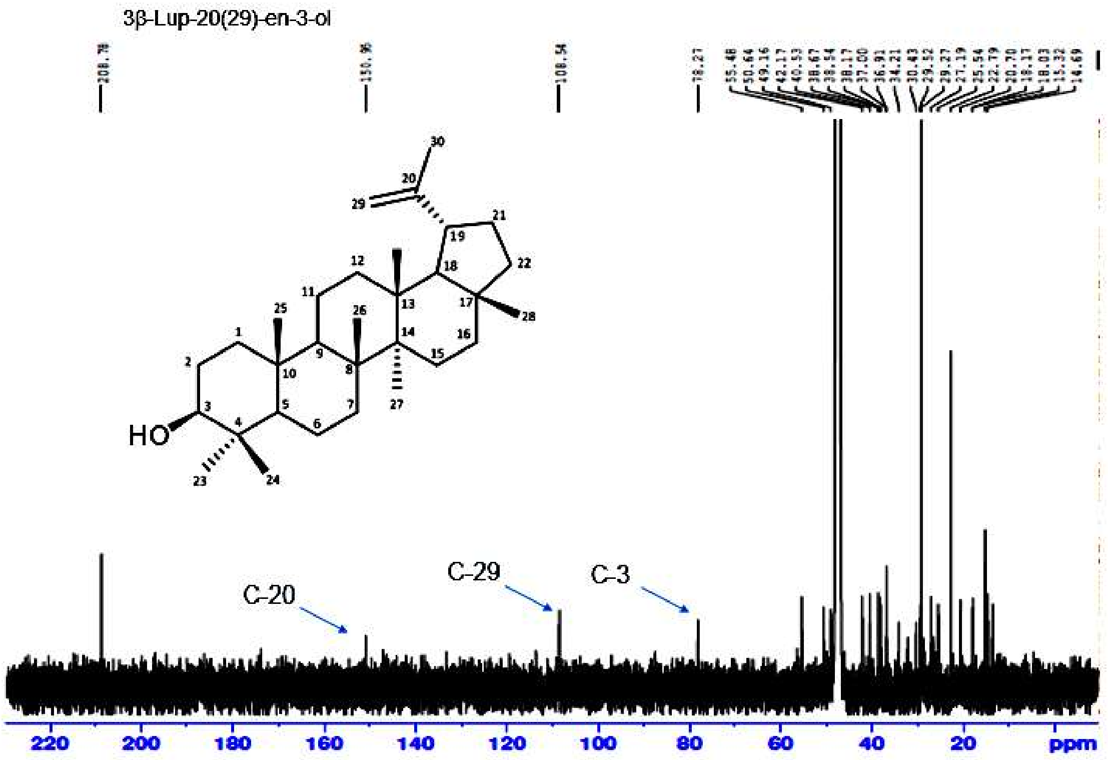

Figure S6. ^{13}C-NMR spectrum of lupeol (**1**).

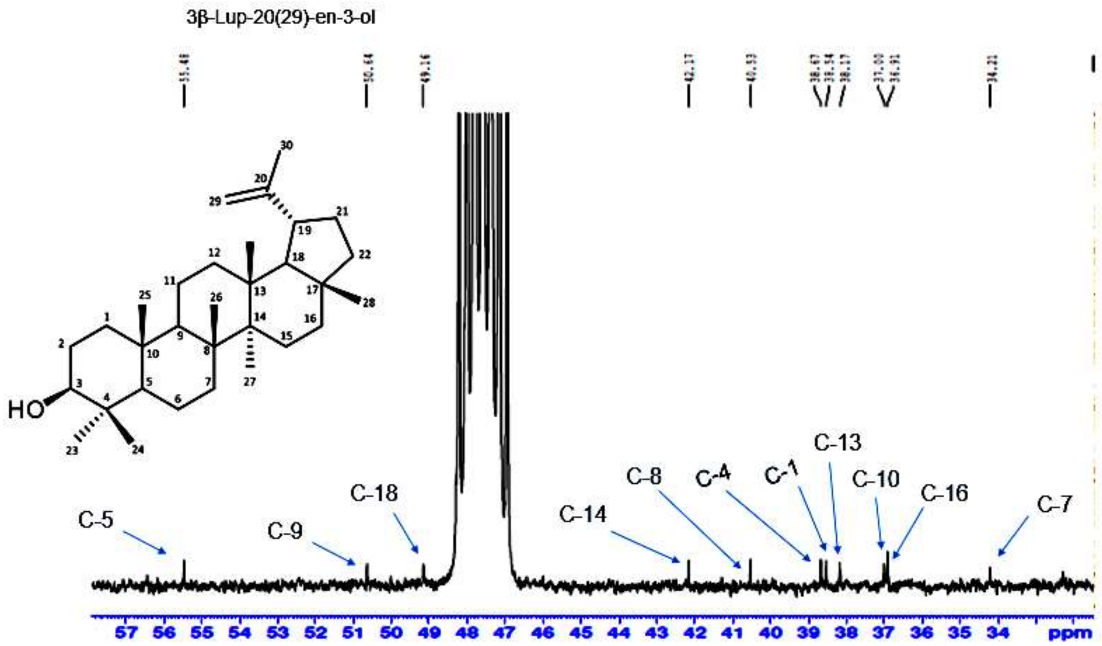

Figure S7. ^{13}C-NMR spectrum of lupeol (**1**).

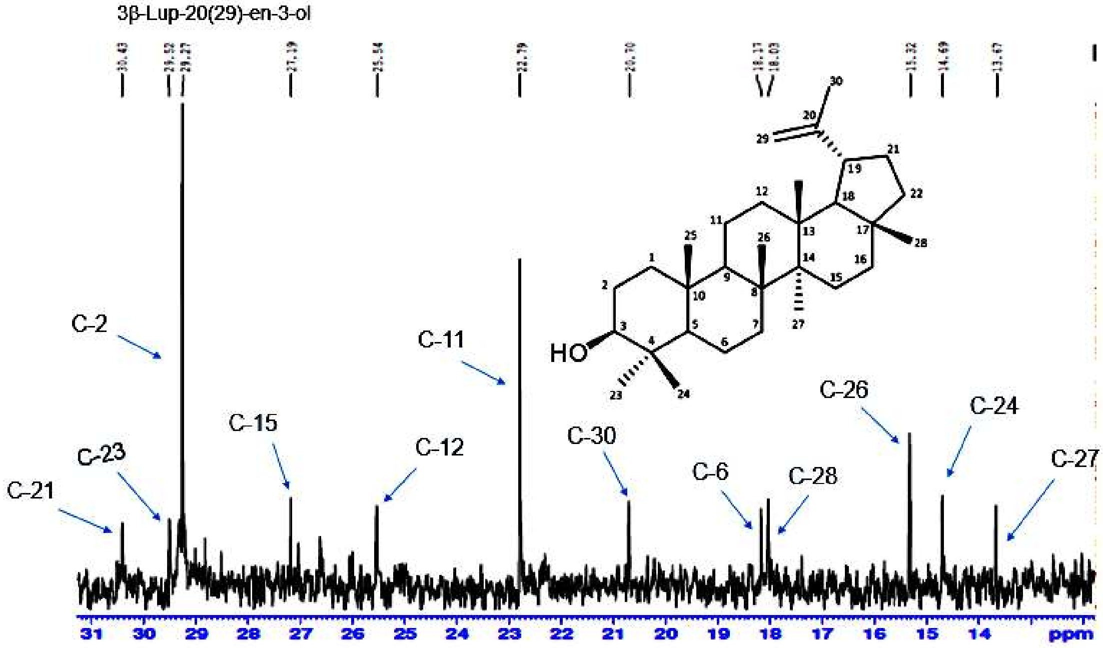

Figure S8. ^{13}C-NMR spectrum of lupeol (**1**).

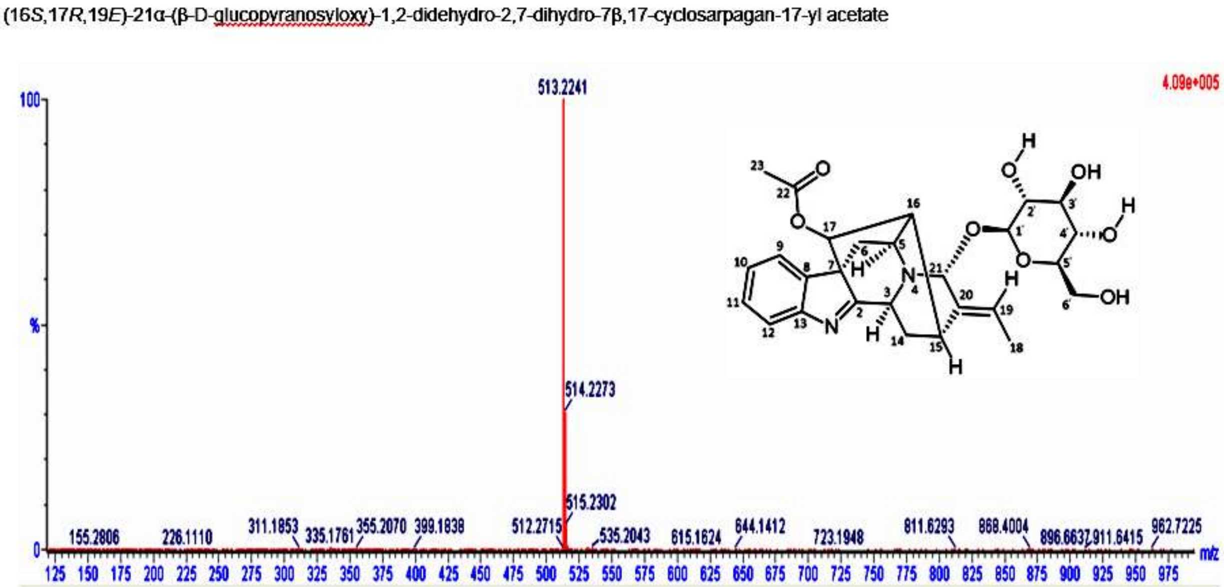

Figure S9. Mass spectrum of raucaffricine (**2**).

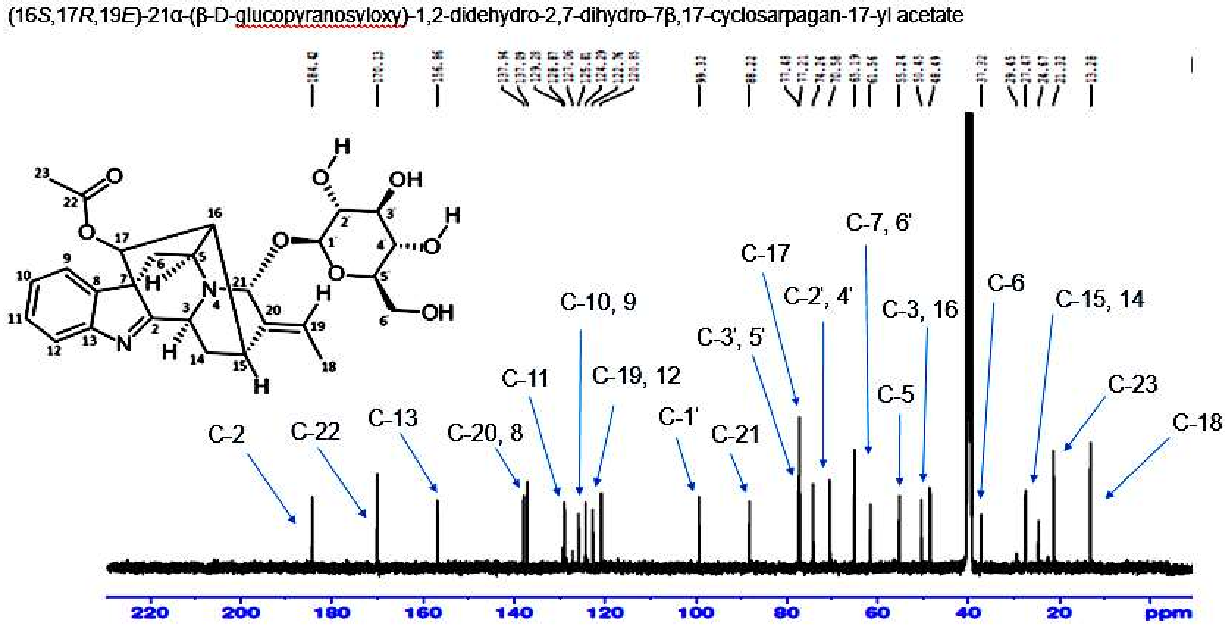

Figure S10. ^{13}C-NMR spectrum of raucaffricine (**2**).

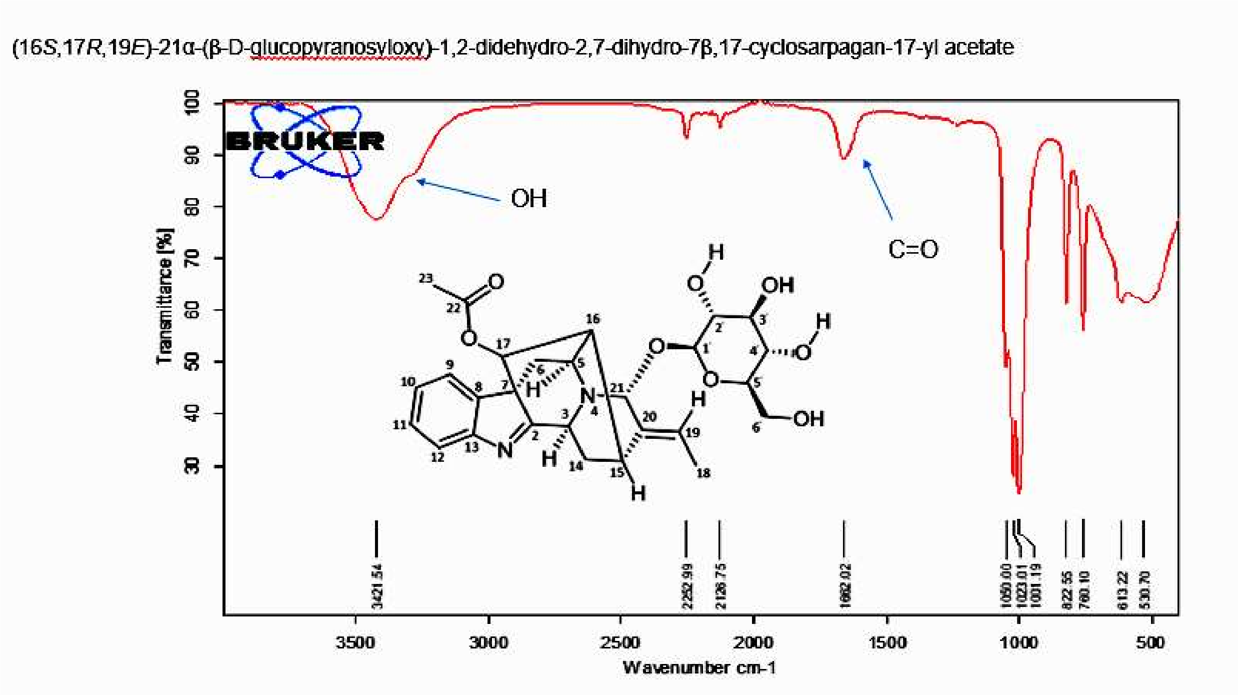

Figure S11. IR spectrum of raucaffricine (**2**).

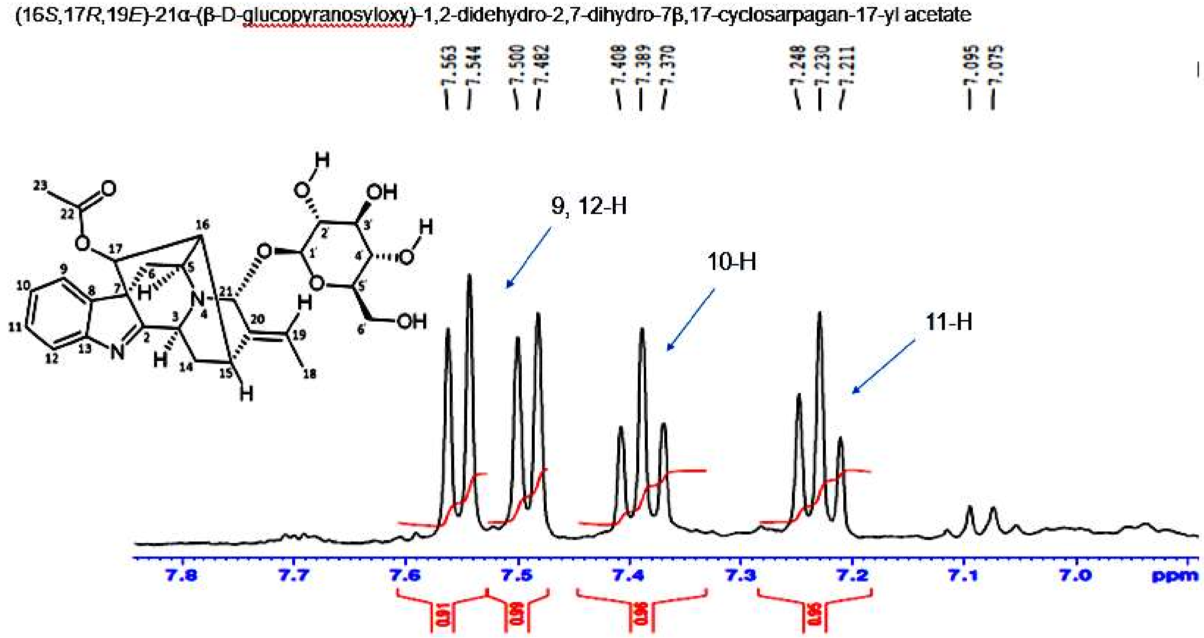

Figure S12. Expanded ^{1}H-NMR spectrum of raucaffricine (**2**).

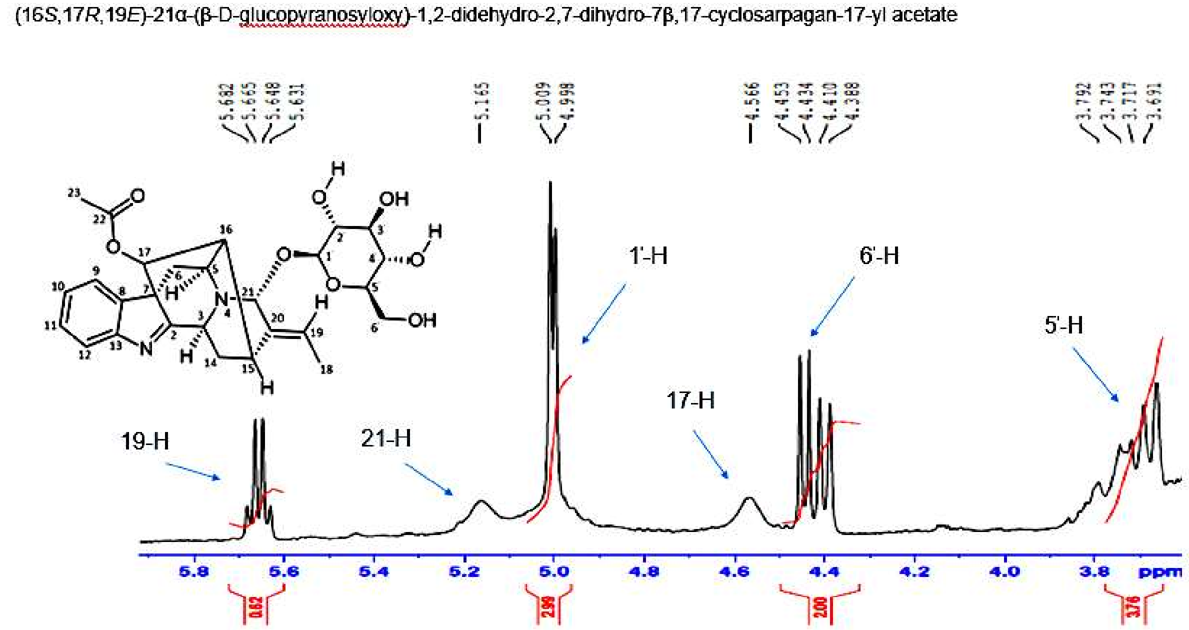

Figure S13. Expanded ^{1}H-NMR spectrum of raucaffricine (**2**).

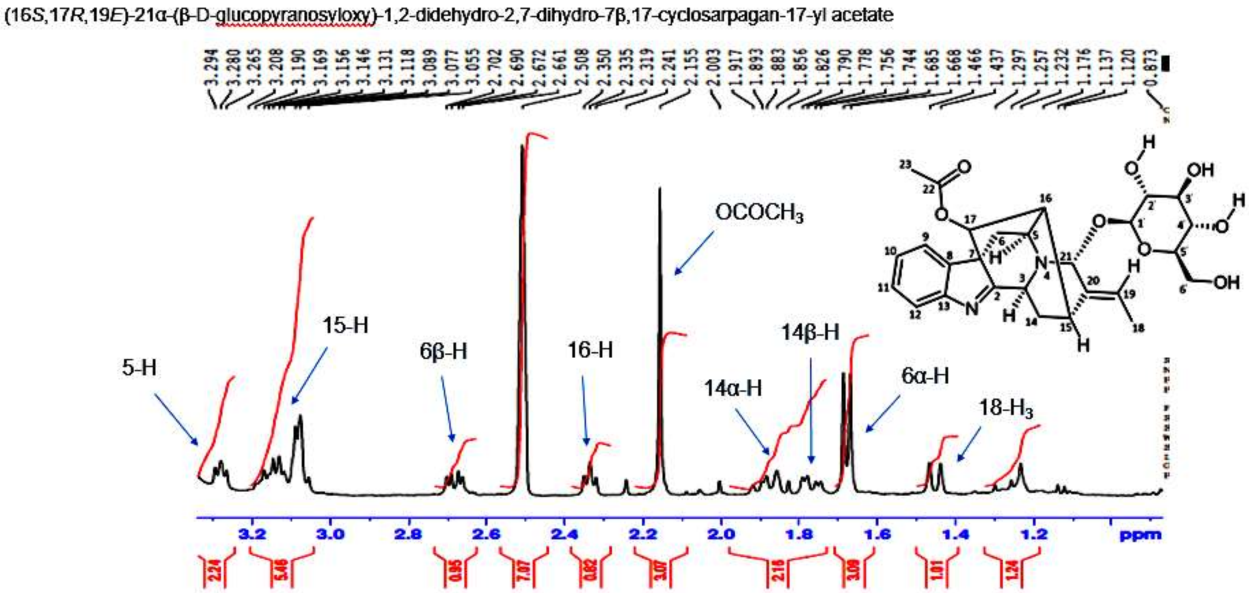

Figure S14. Expanded ^{1}H-NMR spectrum of raucaffricine (**2**).

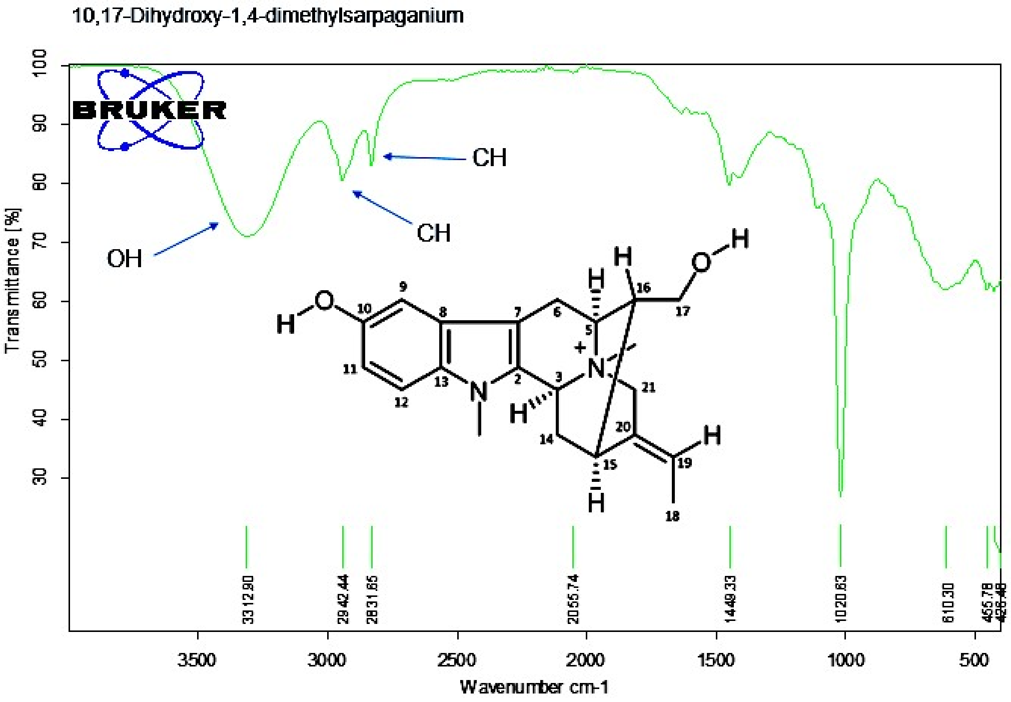

Figure S15. IR spectrum of *N*-methylsarpagine (**3**).

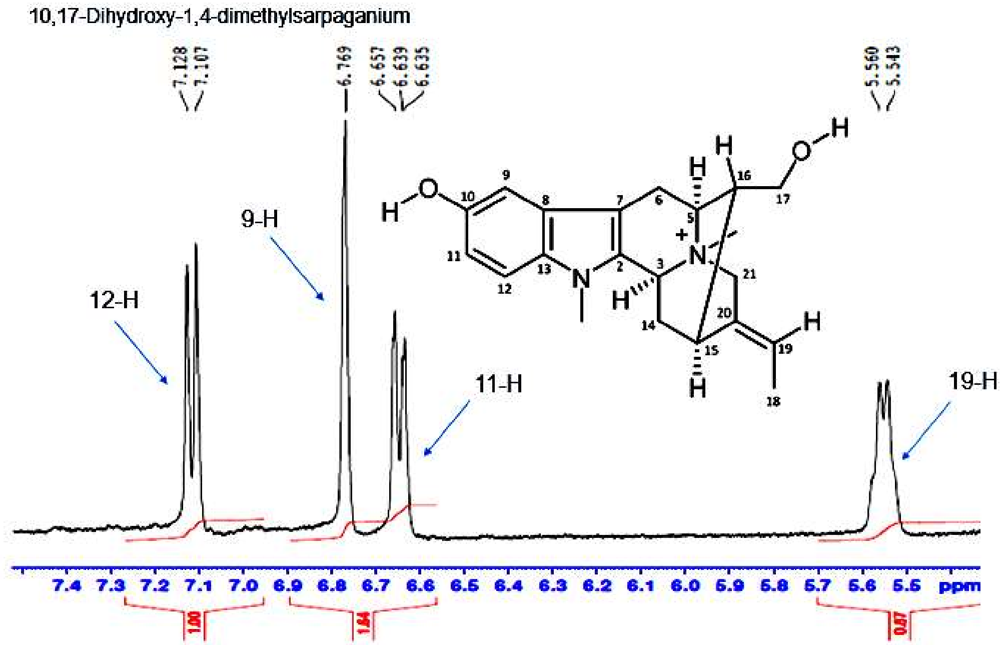

Figure S16. Expanded ^{1}H-NMR spectrum of *N*-methylsarpagine (**3**).

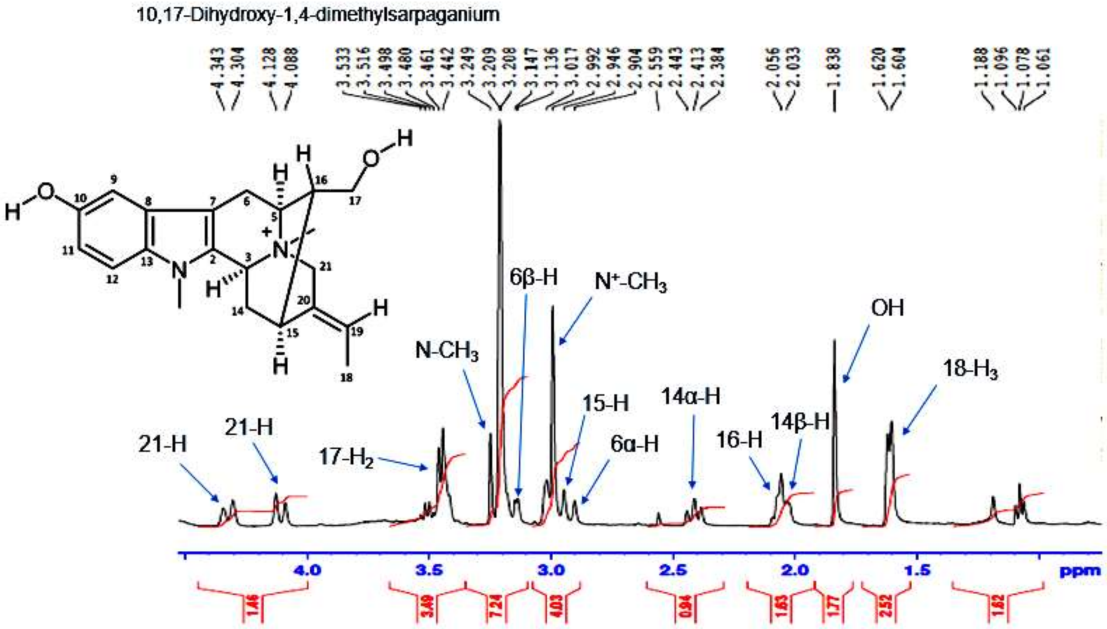

Figure S17. Expanded ^{1}H-NMR spectrum of *N*-methylsarpagine (**3**).

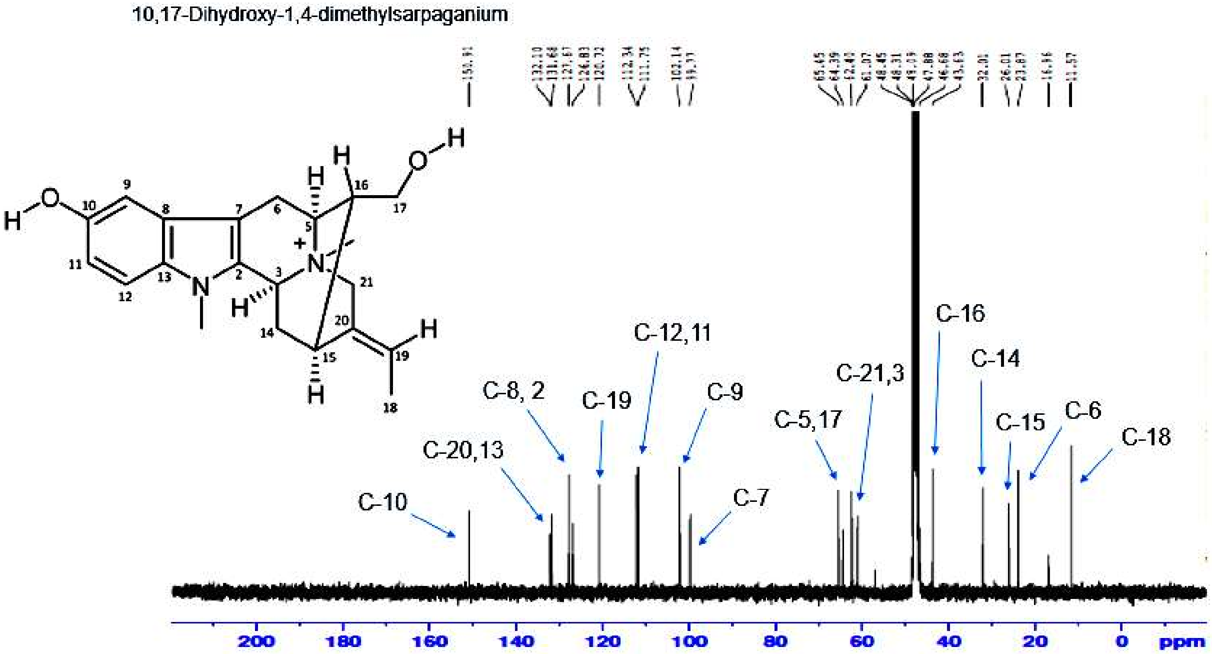

Figure S18. ^{13}C-NMR spectrum of *N*-methylsarpagine (**3**).

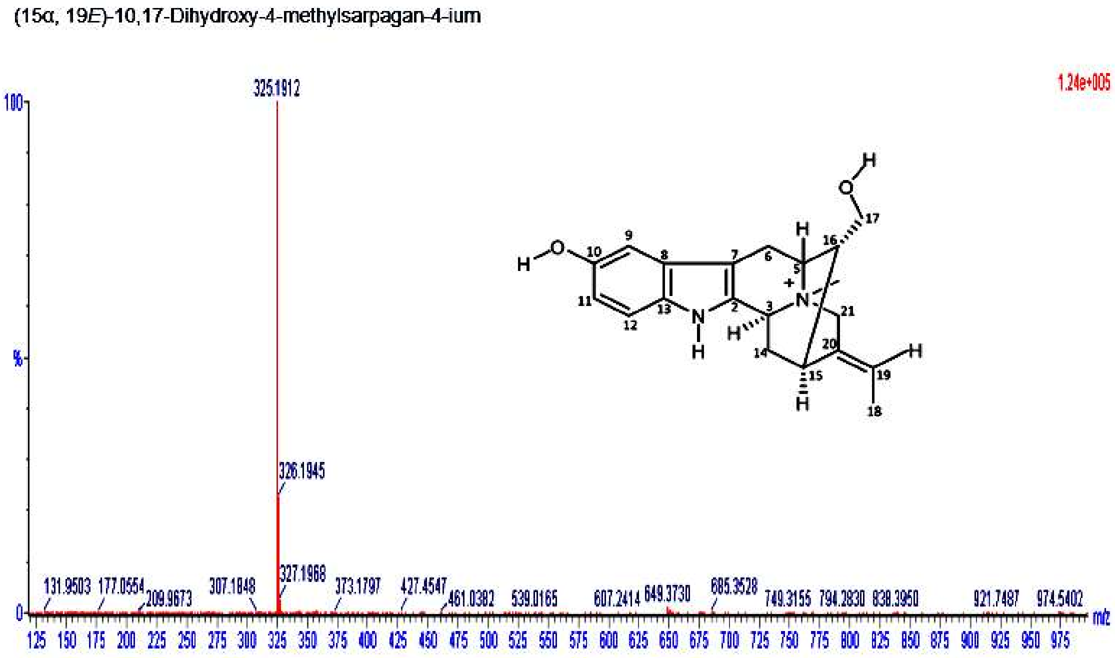

Figure S19. Mass spectrum of spegatrine (**4**).

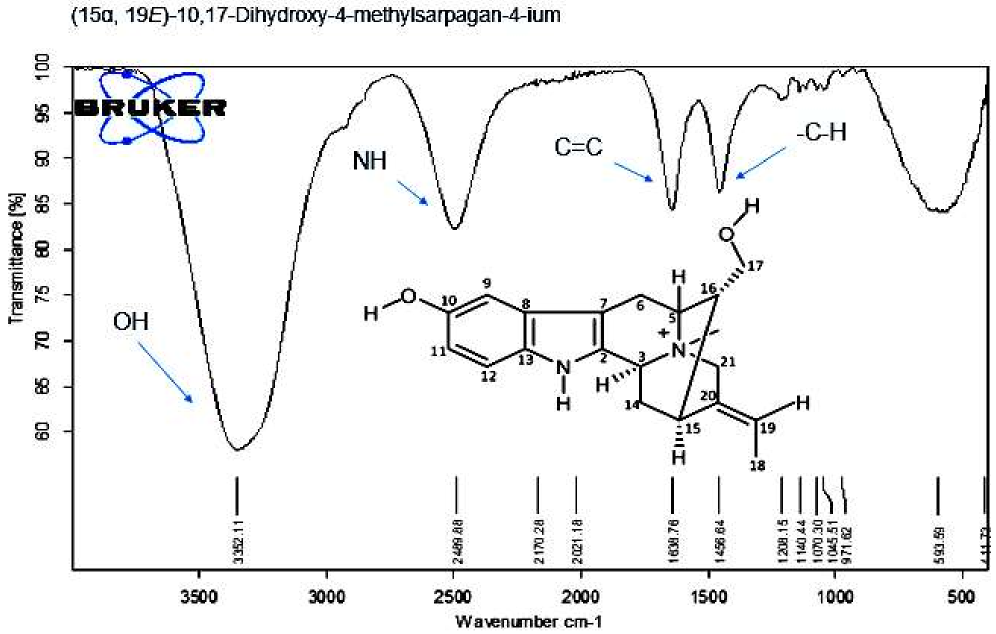

Figure S20. IR spectrum of spegatrine (**4**).

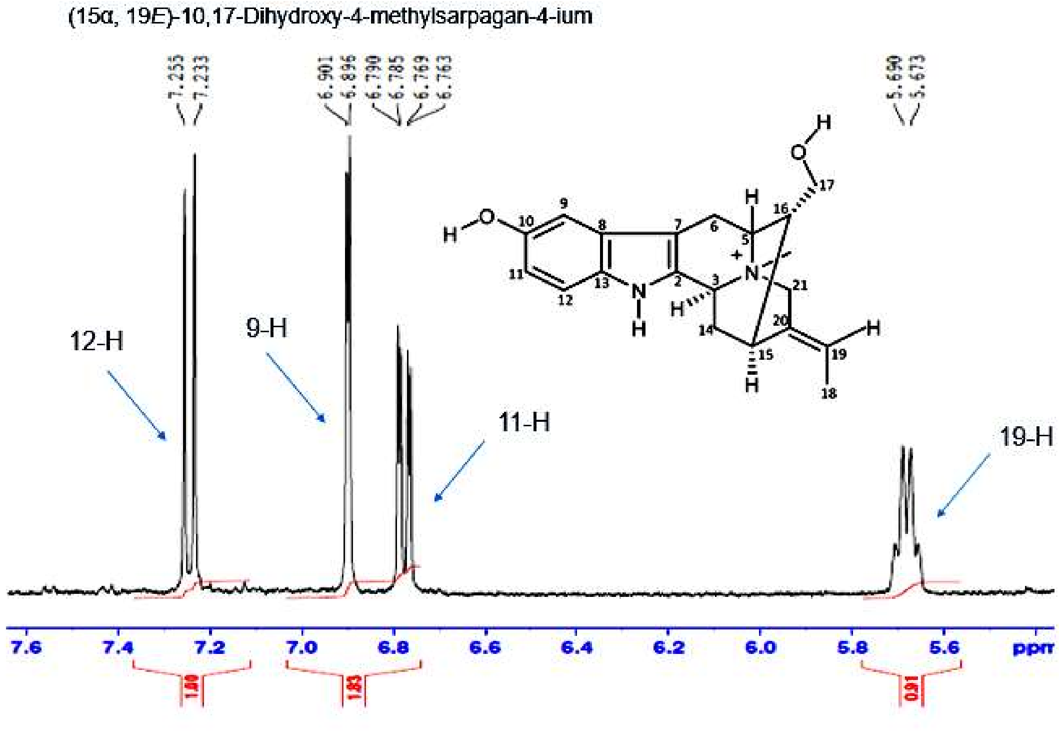

Figure S21. Expanded ^{1}H-NMR spectrum of spegatrine (**4**).

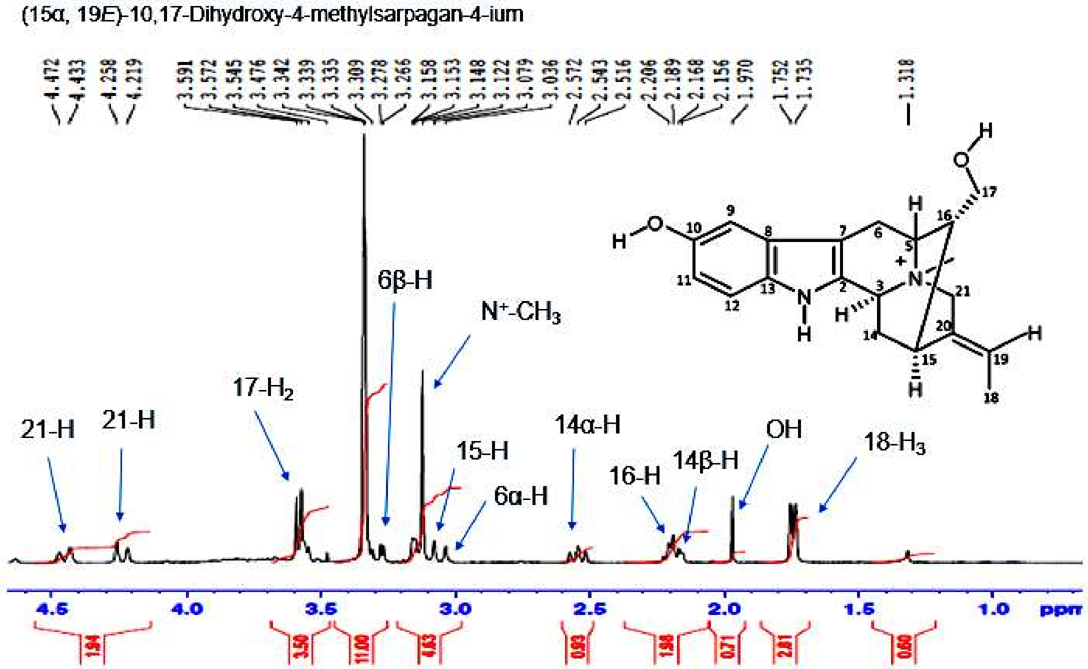

Figure S22. Expanded ^{1}H-NMR spectrum of spegatrine (**4**).

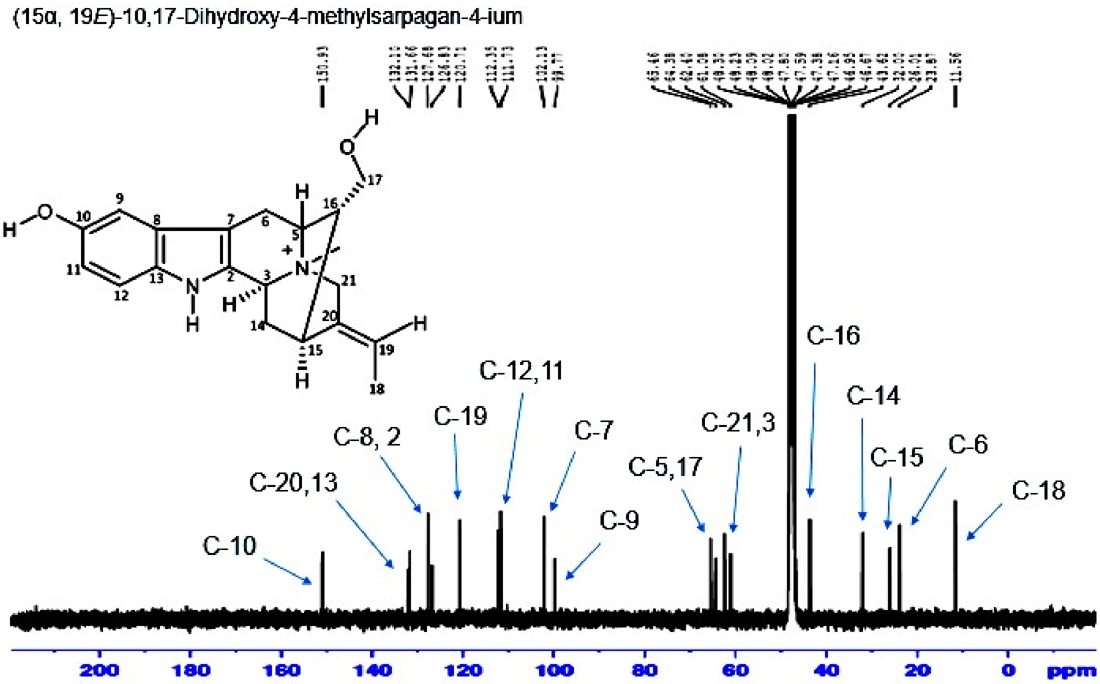

Figure S23. ^{13}C-NMR spectrum of spegatrine (**4**).
